# Race/ethnicity differences in risk and protective factors for marijuana use among U.S. adolescents

**DOI:** 10.1186/s12889-021-11159-z

**Published:** 2021-07-01

**Authors:** Meen Hye Lee, Yeoun Soo Kim-Godwin, Hyungjo Hur

**Affiliations:** 1 School of Nursing, University of North Carolina Wilmington, 601 S College Rd, Wilmington, NC 28403 USA; 2 Department of Public Administration, Dankook University, 152 Jukjeon-ro, Suji-gu, Yongin-si, Gyeonggi-do South Korea

**Keywords:** Marijuana use, Adolescents, Risk factors, Protective factors, Race/ethnicity

## Abstract

**Background:**

Little is known about how race and ethnicity influence marijuana-specific risk and protective factors in U.S. adolescents. We examined differences in risk and protective factors of marijuana use (MU) and their associations with MU by race/ethnicity.

**Methods:**

The present study used data from the 2015–2019 National Survey on Drug Use and Health. A total of 68,263 adolescents (aged 12 to 17 years) were divided into seven subgroups by race/ethnicity (White, Hispanic, Black, Asian, Native American, Native Hawaiian/Pacific Islander (NH/PI), and mixed race). Marijuana-specific risk and protective factors (RPFs) were examined, including perceived availability of marijuana, adolescents’ perceived risk of MU and perceived disapproval of parents, peers, and close friends. Past-month, past-year, and lifetime MU were used as MU outcomes to examine the associations with RPFs as well as with race/ethnicity.

**Results:**

Overall, 6.85, 12.67, and 15.52% of the sample reported past-month, past-year, and lifetime MU respectively. Weighted adjusted logistic regression analyses revealed that mixed race adolescents reported the greatest perceived availability of marijuana, whereas Black and Asian adolescents had less access compared to White adolescents. The adolescents’ perception of parental disapproval of MU was the lowest for Native American adolescents and highest for Asian adolescents. Mixed race adolescents experienced lower peer and close friend disapproval of MU while Black and Asian adolescents had higher. The MU risk perception was lower in most groups including Black, Hispanic, Native American, and mixed race adolescents, but not in Asian adolescents. Native American adolescents scored the highest on all MU outcomes, whereas Asian adolescents scored the lowest. Perceived availability of marijuana was associated with higher MU in all MU outcomes. Lower disapproval MU perceptions and lower MU risk perceptions were also associated with greater MU.

**Conclusion:**

These findings suggest there is considerable heterogeneity of marijuana risk and protective factors and MU across race/ethnicity among U.S. adolescents.

## Introduction

Marijuana (cannabis) is the most commonly used substance after alcohol among adolescents [1]. The 2019 U.S. National Survey on Drug Use and Health (NSDUH) found that 13.2% (3.3 million) of adolescents aged 12–17 had used marijuana in the previous year, with 1.4 million of those being adolescents who used marijuana for the first time [2]. In 2019, 22% of high school seniors in the U.S. currently used cannabis, and 14% had vaped cannabis in the past month [3]. Marijuana use (MU) among adolescents is a particular concern due to its associated health and educational outcomes. Furthermore, adolescents with early marijuana initiation are likely to have relatively lower educational attainment and develop delinquent behaviors, as well as other behavioral and mental health problems [4–6]. Researchers have found that the early onset of MU, or longer duration of MU, may increase vulnerability to addiction and psychiatric disorders later in life [7–9]. People who begin using marijuana before the age of 18 are four to seven times more likely to develop a marijuana use disorder than adults [10].

Researchers claim adolescents’ substance use may be largely influenced by adolescent’s perceptions of substance use norms. These substance use norms may be acquired through parental substance use perceptions and peer substance use. Marijuana norms specifically may be influenced by community drug use levels, marijuana availability, and adolescents’ MU [9]. Thus, adolescents’ perceived MU norms and perceived marijuana availability may serve as risk and protective factors (RPFs) of adolescent MU. Previous studies focusing on parental substance use perceptions have found that parental disapproval was associated with a decreased likelihood of adolescent MU [11]. In addition, peer influence on adolescent cannabis use was also well reported [12–14]. Evidence suggests that youth may be more likely to use on days when they spent more time with friends who use marijuana, as well as when there was a greater availability of marijuana [15]. Furthermore, proximal reference groups (e.g., close friends) have been associated with adolescents’ intention towards substance use as well [16].

Previous research has also identified differences in perceptions of parental or peer disapproval among race/ethnic groups. For example, Non-Hispanic White and Hispanic adolescents reported greater perceptions of peer substance use compared to other ethnicities [17]. Black and Asian adolescents report significantly more parental disapproval compared to White adolescents [18]. In addition, individuals who perceived marijuana as less risky were more likely to report MU than their counterparts who perceived marijuana as more risky [19]. These individual cognitive and family risk factors, as well as intentions to engage in substance use and perceived use by peers have been shown to be related to substance use [20]. Thus, further study is warranted to investigate whether these contributing factors are actually associated with MU among adolescents.

Existing literature shows that the prevalence of adolescent MU varies by racial/ethnic groups. With the exception of Asian Americans, non-White ethnic groups (Black, Hispanic, Native-American, and mixed race) tend to report greater likelihoods of MU than White adolescents [9, 21]. Although a disproportionate amount of adolescent MU between racial/ethnic minorities has been reported, little is known about possible differences in RPFs and their associations with MU. Although Wu et al.’s (2015) study addressed differences in personal, peer, and parental disapproval using 2004–2012 NSDUH data [9], the newly added risk factors (perceived availability of marijuana and adolescents’ MU risk perception) were only considered later than 2015 and not included in the study. In addition, much of the research on ethnic differences tend to categorize racial groups into the standard broad umbrella groups (e.g., White, Hispanic, Black, and Asian) without considering the extensive heterogeneity of people within these categories [22]. This categorization may overlook differences in the examination of social, biological, and cultural factors that may be contributing to the heterogeneity of risk for substance use by non-racial characteristics [20]. In conjunction with the rapidly growing minority population in the U.S. [23], the need for increased understanding warrants an exploration of differences across adolescent racial/ethnic groups. Failure to consider variations in identifying RPFs of MU across racial/ethnic subgroups is a limitation of efforts to examine risk for marijuana use [20]. Thus, using the 2015–2019 NSDUH data, the present study examined differences in marijuana-specific RPFs for MU by race/ethnicity among U.S. adolescents. In addition, this study aimed to investigate whether the marijuana-specific RPFs are linked to MU, as well as the association by race/ethnic differences with MU.

## Method

### Data source

We conducted secondary data analysis using the data gathered from the 2015 to 2019 National Surveys on Drug and Health (NSDUH). The annual survey is designed to provide a nationally representative sample of a non-institutionalized U.S. population aged 12 years or older (SAMHSA, 2019). In this study, adolescents aged 12 to 17 years were included in the analysis (*N* = 68,263). Because previous studies report that age, gender, poverty level, and urbanicity are associated with MU among adolescents [9, 24], logistic regression analyses were conducted controlling for these factors using sampling weights provided by NSDUH. The study was reviewed by the Institutional Review Board of a state university in the southeastern U. S (University of North Carolina Wilmington) which granted a waiver of Institutional Review Board approval.

### Measures

#### Past-month marijuana use

In this study, past-month MU refers to those who used marijuana or hashish within the past 30 days. Respondents self-reported their past-month MU to the question “How long has it been since you last used marijuana or hashish?” If they said yes on the response ‘Within the past 30 days’, we coded 1 and for those who answered ‘Never used or used but more than 30 days ago’, we coded 0.

#### Past-year marijuana use

Past-year MU refers to those who used marijuana or hashish within the past-year. Respondents were asked, “Did you use marijuana or hashish, even once during the past-year?” If they said ‘yes’, we coded 1, and others (‘did not use in the past-year’) 0.

#### Lifetime marijuana use

In the current study, lifetime MU refers to those who self-reported a positive response to the question, “Have you ever, even once, used marijuana or hashish?” We coded 1 if respondents reported ‘yes’, and others (reported ‘no’) 0.

#### Marijuana-specific risk and protective factors

The 2015–2019 NSDUH data used consistent assessments to evaluate five marijuana-specific RPFs based on adolescents’ perceptions of (a) perceived availability of marijuana, (b) parental disapproval of MU, (c) peers’ disapproval of MU, (d) close friends’ disapproval of MU, and (e) risk perception of MU. Each of these items was dichotomized. For perceived availability of marijuana, adolescents were asked, “How difficult or easy would it be for you to get some marijuana if you want some?” A 0 code indicated ‘fairly/very difficult or probably impossible’ and a 1 indicated ‘fairly/very easy’. For parental/peer/close friend disapproval perceptions of MU, we used the question “How do you think (or feel) your parents/someone your age/close friends would feel about you trying marijuana or hashish once or twice?” Responses were coded as 0 indicating ‘neither approve nor disapprove’, or 1 indicating ‘somewhat/strongly disapprove’. Regarding risk perception of MU, each adolescent was asked, “How much do people risk harming themselves physically and in other ways when they smoke marijuana once or twice a week?” (0 = no/slightly/moderate risk, 1 = great risk).

#### Demographics

Self-reported race/ethnicity was assessed respectively. The NSDUH classified seven mutually exclusive racial/ethnic groups: non-Hispanic White, Hispanic, Black, Asian, Native-American, Native Hawaiian/Pacific Islander (NH/PI), and mixed race.

We included sociodemographic information as gender, poverty level (less than 100% of the federal poverty level [FPL], 100 to 199% of the FPL, and 200% or greater); urbanicity: large metro (located in a population segment in a core-based statistical area [CBSA] with 1 million or more persons), small metro (located in a population segment in a CBSA with fewer than 1 million persons), or non-metro (located in a population segment not in a CBSA); and age categorized into three groups (12–13,14–15, and 16–17 years), and survey years to lessen for their confounding effects on estimated associations. Weighted descriptive statistics are reported in Table 1. For all analyses, we used Stata version 15.

**Table 1:** Descriptive Statistics of the sample (*n* = 68,263)

Variables	Group	Percentage
Race	White	52.62%
	Black	13.64%
	Hispanic	23.98%
	Asian	5.37%
	Native Hawaiians/Pacific Islander	0.44%
	Native-Americans	0.63%
	Mixed-race	3.32%
Gender	Male	50.94%
	Female	49.06%
Poverty level	< 100%	21.69%
	100–199%	21.80%
	200%+	56.51%
Urbanicity	Large metro	54.39%
	Small metro	39.77%
	Non-metro	5.84%
Age	12–13	31.58%
	14–15	34.32%
	16–17	34.10%
Perceived availability of marijuana	Yes	45.61%
Parental disapproval of marijuana use	Yes	94.16%
Peer disapproval of marijuana use	Yes	78.92%
Close friends disapproval of marijuana use	Yes	78.28%
Risk perception of marijuana use	Yes	37.29%
Past month marijuana use	Yes	6.85%
Past year marijuana use	Yes	12.67%
Lifetime marijuana use	Yes	15.52%

## Results

Table 1 describes the demographics and weighted prevalence estimates of MU for the sample. Overall, White adolescents represented about half (52.62%) of the sample followed by Hispanic adolescents (23.98%), Black adolescents (13.64%), Asian adolescents (5.37%), mixed race (3.32%), Native American adolescents (0.63%), and Native Hawaiian/Pacific Islander adolescents (0.44%). Of the respondents, 50.94% were male, with 43.49% living at less than 200% of the federal poverty level. In addition, 94.16% of the adolescents resided in large metro or small metro areas. Responses from the total sample indicated 6.85% past-month MU, 12.67% past-year MU, and 15.52% lifetime MU. 45.61% of adolescents reported positive responses on perceived availability of marijuana. Parental disapproval of MU was the most prevalent among the sample (94.16%), followed by peer disapproval (78.92%) of MU, and close friend disapproval (78.28%). 37.29% of adolescents reported perceived great risk of MU.

Table 2 provides weighted logistic regression estimates of differences in marijuana-specific RPFs across race/ethnicity controlling for confounders. White adolescents served as a reference group on race/ethnicity in all logistic regression analyses. Mixed race adolescents reported the greatest likelihood of perceived availability of marijuana (OR: 1.24, 95% CI: 1.12–1.36) while Asian adolescents reported the lowest perceived availability of marijuana (OR: 0.46, 95% CI: 0.41–0.53). Regarding the perceptions of parental/peer/close friends’ disapproval of MU, the odds of disapproval by a peer, parents, and close friend were higher for Asian adolescents compared with White adolescents (OR ranging from 2.00 to 2.89). Hispanic adolescents had higher odds of disapproval by parents (OR: 1.46, 95% CI: 1.29–1.64) and close friends (OR: 1.10, 95% CI: 1.02–1.18), and Black adolescents had higher odds of disapproval by a peer (OR: 1.15, 95% CI: 1.06–1.25). Native American adolescents reported the lowest odd of parental disapproval (OR: 0.68, 95% CI: 0.50–0.91). With respect to risk perception of MU, Native American adolescents reported the lowest odds (OR: 0.58, 95% CI: 0.47–0.71), followed by mixed race adolescents (OR: 0.67, 95% CI: 0.61–0.74). Asian adolescents, on the other hand, had the lowest perceived availability of marijuana and the highest risk perception of MU among all groups (OR: 1.69, 95% CI: 1.50–1.91).

**Table 2:** Adjusted odds ratio (AOR) of marijuana-specific risk and protective factors among U.S. adolescents

	Perceived availability of marijuana (*n* = 66,019)	Parental disapproval of marijuana use (*n* = 67,111)	Peer disapproval of marijuana use (*n* = 67,187)	Close friends disapproval of marijuana use (*n* = 66,920)	Risk perception of marijuana use (*n* = 66,825)
Race (Ref: White)					
Black	0.807***	1.079	1.151**	1.074	0.787***
	(0.750–0.868)	(0.963–1.208)	(1.057–1.253)	(0.988–1.166)	(0.738–0.840)
Hispanic	1.014	1.455***	1.068	1.099*	0.903***
	(0.949–1.083)	(1.294–1.636)	(0.984–1.159)	(1.022–1.182)	(0.854–0.955)
Asian	0.464***	2.885***	1.996***	2.491***	1.690***
	(0.406–0.530)	(2.048–4.063)	(1.747–2.281)	(2.244–2.766)	(1.497–1.907)
Native Hawaiians/Pacific Islander	0.957 (0.678–1.352)	0.694 (0.372–1.296)	0.825 (0.524–1.300)	0.960 (0.614–1.502)	0.719 (0.483–1.070)
Native-Americans	0.834	0.675*	0.873	0.883	0.579***
	(0.682–1.019)	(0.500–0.910)	(0.691–1.104)	(0.690–1.129)	(0.473–0.708)
Mixed-race	1.236***	0.818	0.765***	0.801***	0.673***
	(1.122–1.361)	(0.660–1.014)	(0.672–0.870)	(0.709–0.904)	(0.611–0.741)
Male (ref: female)	0.800***	1.045	1.061**	0.939**	0.801***
	(0.765–0.835)	(0.952–1.147)	(1.018–1.106)	(0.899–0.981)	(0.770–0.833)
Poverty level (ref: < 100%)					
100–199%	1.239***	1.422***	0.994	0.985	1.134**
	(1.153–1.331)	(1.250–1.619)	(0.908–1.088)	(0.919–1.055)	(1.050–1.225)
200%+	1.414***	1.991***	0.946	0.989	1.290***
	(1.328–1.506)	(1.777–2.231)	(0.882–1.015)	(0.923–1.059)	(1.204–1.382)
Urbanicity (ref: Large metro)					
Small metro	0.954*	0.915	1.080*	1.043	1.012
	(0.911–1.000)	(0.825–1.015)	(1.017–1.147)	(0.989–1.101)	(0.958–1.070)
Non-metro	0.754***	1.100	1.419***	1.374***	1.240***
	(0.688–0.827)	(0.946–1.279)	(1.278–1.575)	(1.232–1.534)	(1.102–1.396)
Age (ref: 12, 13)					
14–15	3.963***	0.590***	0.275***	0.296***	0.519***
	(3.778–4.157)	(0.514–0.676)	(0.249–0.304)	(0.267–0.327)	(0.489–0.552)
16–17	9.317***	0.312***	0.131***	0.133***	0.290***
	(8.720–9.955)	(0.274–0.357)	(0.120–0.144)	(0.120–0.146)	(0.273–0.308)
Year (ref: 2015)					
2016	0.928*	0.869	0.957	1.010	0.982
	(0.865–0.995)	(0.738–1.024)	(0.869–1.054)	(0.923–1.105)	(0.910–1.059)
2017	0.986	0.785***	0.925*	0.917*	0.907*
	(0.927–1.048)	(0.690–0.893)	(0.857–0.998)	(0.850–0.990)	(0.841–0.979)
2018	0.978	0.745***	0.848***	0.847***	0.759***
	(0.898–1.066)	(0.651–0.852)	(0.783–0.918)	(0.780–0.919)	(0.711–0.809)
2019	0.941	0.682***	0.854***	0.860***	0.736***
	(0.866–1.022)	(0.594–0.783)	(0.791–0.923)	(0.796–0.929)	(0.683–0.792)

95% confidence intervals (CI) in parentheses; Ref: Reference group

*** *p* < 0.001, ** *p* < 0.01, * *p* < 0.05

To estimate the associations with MU by race/ethnicity and marijuana-specific RPFs, we conducted a weighted logistic regression with different MU outcomes (past-month MU, past-year MU, and lifetime MU) controlling for confounders. In general, all RPFs were significantly associated with all MU outcomes (Table 3). The perceived availability of marijuana was associated with higher MU. Lower parental/peer/close friends’ disapproval of MU was also associated with higher MU outcomes. Regarding the racial/ethnic differences in MU outcomes, Native American adolescents reported the highest MU (ORs of past-month MU: 3.09, past-year MU: 2.74, and lifetime MU: 2.52), while Asian adolescents reported the lowest MU in past-year and lifetime MU reports (ORs of past-month MU: 0.66, past-year MU: 0.58, and lifetime MU: 0.59). Mixed race adolescents (OR: 1.17, 95% CI: 1.01–1.36), Hispanic adolescents (OR: 1.20, 95% CI: 1.10–1.30), and Black adolescents reported higher lifetime MU (OR: 1.16, 95% CI: 1.05–1.27) than White adolescents.

**Table 3:** Multivariate adjusted logistic regression for variables associated with marijuana use (MU) among U. S adolescents

	Past-month MU (*n* = 64,036)	Past-year MU (*n* = 64,036)	Lifetime MU (*n* = 66,391)
Perceived availability of marijuana	6.257***	4.774***	4.580***
	(5.401–7.250)	(4.362–5.225)	(4.181–5.016)
Parental disapproval of marijuana use	0.477***	0.600***	0.637***
	(0.406–0.560)	(0.524–0.686)	(0.560–0.725)
Peers disapproval of marijuana use	0.355***	0.347***	0.371***
	(0.316–0.399)	(0.314–0.383)	(0.340–0.406)
Close friends disapproval of marijuana use	0.321***	0.343***	0.370***
	(0.281–0.367)	(0.313–0.376)	(0.340–0.402)
Risk perception of marijuana use	0.245***	0.259***	0.288***
	(0.194–0.310)	(0.224–0.300)	(0.255–0.324)
Race and gender			
Black	1.104	1.119	1.156**
(Ref: White)	(0.944–1.291)	(0.996–1.258)	(1.053–1.270)
Hispanic	1.042	1.083	1.200***
	(0.898–1.209)	(0.986–1.191)	(1.109–1.298)
Asian	0.663*	0.580***	0.585***
	(0.459–0.957)	(0.450–0.747)	(0.466–0.735)
Native Hawaiians, Pacific	1.170	0.993	1.123
	(0.514–2.662)	(0.545–1.809)	(0.608–2.077)
Native-Americans	3.088***	2.742***	2.552***
	(2.184–4.367)	(2.013–3.734)	(1.977–3.294)
Mixed-race	1.227*	1.161	1.167*
	(1.051–1.433)	(0.989–1.364)	(1.005–1.356)
Male (ref: female)	1.167**	0.992	1.026
	(1.046–1.301)	(0.916–1.075)	(0.960–1.097)
Poverty level (ref: < 100%)			
100–199%	0.921	0.882**	0.807***
	(0.817–1.038)	(0.809–0.961)	(0.734–0.889)
200%+	0.761***	0.791***	0.638***
	(0.675–0.857)	(0.708–0.883)	(0.575–0.708)
Urbanicity			
Small metro	0.991	1.005	1.077
(ref: Large metro)	(0.904–1.087)	(0.928–1.088)	(0.998–1.163)
Non-metro	0.876	0.961	1.105
	(0.732–1.048)	(0.832–1.110)	(0.936–1.304)
Age (ref: 12, 13)			
14–15	2.421***	2.571***	2.602***
	(1.924–3.045)	(2.264–2.921)	(2.305–2.939)
16–17	3.828***	3.890***	4.626***
	(3.141–4.665)	(3.407–4.442)	(4.068–5.261)
Year (ref: 2015)			
2016	0.843*	0.900*	0.878*
	(0.726–0.980)	(0.814–0.996)	(0.795–0.969)
2017	0.815*	0.912	0.886
	(0.688–0.966)	(0.796–1.045)	(0.780–1.007)
2018	0.787**	0.858**	0.821***
	(0.676–0.916)	(0.767–0.961)	(0.737–0.914)
2019	1.036	1.044	0.970
	0.843*	(0.917–1.188)	(0.876–1.075)

95% confidence intervals (CI) in parentheses; Ref: Reference group

*** *p* < 0.001, ** *p* < 0.01, * *p* < 0.05

## Discussion

The present study examined the differences and associations in marijuana-specific RPFs for MU among US adolescents. Overall, the relationships between RPFs and MU outcomes were in the expected direction. Perceived availability of marijuana was positively related to past-month, past-year, and lifetime MU. In contrast, risk perception and parental/peer/friend disapproval were negatively related to current, past-year, and lifetime MU. In particular, perceived availability of marijuana had stronger magnitudes with past-month MU (OR = 6.26) compared to past-year MU (OR = 4.77) and lifetime MU (OR = 4.58). Previous studies reported that the greater availability of illicit drugs predicts higher levels of drug initiation and use [18, 22, 24]. In contrast, risk perception of MU was negatively associated with past-month, past-year, and lifetime MU, supporting the previous studies that a low perceived risk of MU was the contributing factor promoting adolescent MU [8, 13]. Considering the gradual increase of recreational marijuana legalization in the US and general acceptance of MU among adolescents [17, 25], policymakers should establish effective surveillance policies to reduce marijuana accessibility and health educators should increase effective education programs for adolescents.

Disapproval of parent/peer/friend was inversely related to past-month, past-year, and lifetime MU in the present study. Previous studies have shown that parenting is associated with substance use among adolescents. For example, authoritative parenting was associated with lower use of alcohol, tobacco, and illicit drugs in children and adolescents [26, 27], and neglectful parenting style was associated with worse substance use outcomes [28]. Ruybal and Crano (2020) reported that parental monitoring was associated with an increased likelihood of MU among US adolescents with depressive symptoms [6]. Emerging evidence also suggests that parental education and training that focuses on diverse messages, such as risk communication and providing factual information about the law, would be helpful in preventing adolescent MU [21].

Both disapproval from peers and close friends were strongly associated with adolescent MU, consistent with the previous reports [17, 29]. There may be a powerful influence from an adolescent’s peers providing substance access and active encouragement or discouragement of use, which may strongly shape substance use behavior [14]. While both disapproval from peers and close friends were strongly associated with adolescent MU, the impact can be greatly influenced by the level of the friendship. This is because not all kinds of peers bring the same power of peer pressure. Previous studies found closer friendships inflict higher levels of influence than acquaintances [16, 30]. For example, in scenarios involving alcohol, more people tried substances when gathering with close friends compared to when attending large parties filled with strangers. Even though peer pressure exists in scenarios involving more acquaintances and strangers than close friends, this impact was magnified as the magnitude of the friendship increased [30]. Furthermore, Schuler et al. (2019) analyzed data from five longitudinal surveys from a cohort of youth (grades of 6–12), finding that best friend substance use was the stronger predictor of substance use than family factors [14].

Reaffirming the RPFs of MU validates many prevention and intervention programs that focus on peer and parental influences on substance use. For example, the I Can Problem Solve (ICPS) program [31] is designed to enhance skills associated with critical thinking and decision making, which has been shown to be associated with characteristics related to later onset substance abuse (i.e., school bonding, social competence, self-regulation) [32]. This program proved that utilizing both of these programs focusing broadly on the school environment and the family environment among elementary school-aged children was effective in improving all characteristics associated with later onset substance abuse, including school bonding, parenting skills, social competence, family relationships, and self-regulation compared to elementary school-aged children exposed to the ICPS program alone as well as a control group that did not receive either intervention [32]. These findings support the efficacy of combining programs focusing on characteristics related to the broader realms of the school, where youth may be susceptible to peer influence, and family environment, where youth may be susceptible to parental influence, being that both of these realms can influence characteristics related to the later onset of substance abuse [32].

Overall, these marijuana-specific RPFs varied remarkably by race/ethnicity were associated with MU. Out of all ethnic groups, Asian adolescents showed the lowest past-year and lifetime MU. The 2012 national study among youth 12–17 also showed that Asian Americans were approximately twice as likely as White adolescents to report personal, parental, or close friend disapproval of cannabis use [33]. The lower MU could be partially explained by Asian adolescents having remarkably lower access and higher risk perception, as well as the highest disapproval from parents/peer/close friends. Parent–youth relationship and parental attitudes toward substance use are significant substance use correlates among Asian American youth [34]. Asian Americans may tend to endorse collectivistic parenting, while Caucasian parents tend to endorse individualistic parenting styles [35]. Authoritarian parenting, which encompasses low parental warmth and high parental control, is common in Asian families [34]. Although an authoritarian parenting style is considered less than optimal in individualistic Western cultures, it has been associated with academic achievement and social-emotional adjustment in collectivistic Asian cultures [36].

While the current study showed that Hispanic adolescents reported higher lifetime MU than White adolescents, the reason for this finding is not clear. One possible explanation involves a lower risk perception of MU for Hispanic adolescents despite a higher level of parental and friend marijuana disapproval. Merianos and colleagues (2020), who conducted an analysis of the 2012 NSDUH for Hispanic adolescents, reported that most authoritative parenting behaviors have a significant effect against MU. Moreover, Hispanic culture places an emphasis on family values, which may act as a protective factor in adolescent substance use [34]. The study findings suggest that intervention programs specifically designed for Hispanic adolescents may need to place an emphasis on involving parents in marijuana prevention programs and incorporate education on how parenting styles are associated with adolescents’ MU, being that the family may be a more important protective factor for this ethnic group compared to others.

The current study showed Black adolescents reported higher lifetime MU in spite of a comparatively lower perceived access and higher peer disapproval of marijuana than White adolescents. However, this higher lifetime MU may perhaps be explained by a lower risk perception of MU for Black adolescents. There have been notable rises in marijuana use disorder among Black and Hispanic adolescents compared to White adolescents, which may also partially be explained by a decrease in risk perceptions of MU [37]. Research focusing on additional rationales for higher lifetime MU among Black adolescents is warranted.

Out of all groups studied, the Native American (NA) group had the lowest risk perception and parental disapproval, which may explain why this group has the highest past-month, past-year, and lifetime MU. Earlier studies showed that the availability of drugs was associated with drug initiation and use, reporting higher prevalence of adolescents’ MU among NA groups [9, 21, 25]. The current study showed NA had the highest rates by far in all three marijuana use outcomes. Such findings were unsurprising, given the low NA risk perception and the previous literature revealing perceived risks to be strong predictors of MU for NA adolescents [38]. Spillane and colleagues (2021) reported that among the sample of NA 7th–12th graders in 2009–2013, about 60% reported lifetime MU and over two-thirds reported that it would be ‘fairly easy’ or ‘very easy’ to get marijuana. The findings of the study indicated that perceived marijuana availability was associated with greater acceptability (i.e., less perceived risks) and greater perceived approval (i.e., decreased injunctive norms) of MU [39]. In fact, easy access to marijuana has been identified as among the strongest predictors of adolescent MU [40]. The present study findings imply that NA adolescents are at risk of high exposure to MU in their youth.

As mentioned previously, the racial/ethnic differences found in the current study among marijuana-specific RPFs and their association with MU are important for designing and implementing prevention and intervention programs for at-risk minority youth. Research has shown that in order for prevention and intervention programs to be effective across multiple racial/ethnic groups, they must be culturally sensitive [41]. The current study supports this, in that RPFs of MU further differ among racial/ethnic subpopulations. Thus, identifying that RPFs of MU do differ among racial/ethnic subpopulations is one of the first steps in designing and implementing intervention and prevention programs that are culturally sensitive, which may be increasing their effectiveness for at-risk minority youth.

### Limitations

The present study has several limitations. First, our study used a relatively small sample size of Native Hawaiians/Pacific Islander and Native American adolescents for race comparison in the analyses. However, we used survey weights provided by NSDUH in our analyses to improve the generalizability of our estimates. Second, our study used the NSDUH cross-sectional design data, so causality could not be determined. Longitudinal studies could better explain how RPFs predict co-use of marijuana with other substances by their age (such as early and late adolescence) in young adulthood [29]. As such, more longitudinal research on this topic is needed to comprehensively understand the complexity of MU across race/ethnicity groups over time. Despite these limitations, this study enhances understanding of MU in adolescents by revealing the considerable heterogeneity of marijuana RPFs across race/ethnicity among US adolescents.

## Conclusion

The findings of the study underscore the importance of RPFs in past-month, past-year, and lifetime MU among US adolescents. Concerning adolescents’ perception of MU disapproval of parents, peers, and close friends, the present study showed considerable heterogeneity between races/ethnicities. Engaging parental support and establishing ethnic community involvement could further decrease the likelihood of MU. In addition, policymakers and school health educators should consider the importance of peer pressure on MU, thereby coordinating school environments to alleviate this pressure.

Identifying marijuana RPFs is crucial to preventing and reducing the continuation of MU among adolescents. This study highlights the varied RPFs on past-month, past-year, and lifetime MU across races/ethnicities. Furthermore, this study helps to explain racial/ethnic disparities in MU among adolescents. Racial differences suggest the potential utility of culturally tailored interventions that focus on differences in RPFs of MU. It is therefore imperative to examine any variations in risk that may be attributed to race/ethnicity, as there are significant differences across the groups, which may have noteworthy implications for the treatment of substance use disorders and comorbid problems [42]. In particular, extra attention on Native American individuals to prevent MU is warranted from the current findings. Prevention programs aimed at reducing potential harm from MU being essential for this group, as well as other racial/ethnic groups. These prevention programs are arguably urgent, being that the spread of the legalization of marijuana in the United States could play a major role in risk perceptions in the future. Further policy and the development of prevention programs reflecting cultural characteristics of each racial/ethnic group are recommended.

## Data Availability

The datasets generated and/or analyzed during the current study are available from the corresponding author on reasonable request.
